# Estimating the effect of focused donor registration efforts on the number of organ donors

**DOI:** 10.1371/journal.pone.0241672

**Published:** 2020-11-04

**Authors:** James H. Cardon, Jordan C. Holbrook, Mark H. Showalter

**Affiliations:** 1 Department of Economics, Brigham Young University, Provo, UT, United States of America; 2 Department of Economics, University of Houston, Houston, TX, United States of America; Imperial College Healthcare NHS Trust, UNITED KINGDOM

## Abstract

Waiting times for organs in the United States are long and vary widely across regions. Donor registration can increase the number of potential donors, but its effect on the actual number of organ transplants depends upon several factors. First among these factors is that deceased donor organ donation requires both that death occur in a way making recovery possible and that authorization to recover organs is obtained. We estimate the potential donor death rate and donor authorization rate conditional on potential donor death by donor registration status for each state and for key demographic groups. With this information, we then develop a simple measure of the value of a new donor registration. This combined measure using information on donor authorization rates and potential death rates varies widely across states and groups, suggesting that focusing registration efforts on high-value groups and locations can significantly increase the overall number of donors. Targeting high-value states raises 26.7 percent more donors than a uniform, nationwide registration effort. Our estimates can also be used to assess alternative, but complementary, policies such as protocols to improve authorization rates for non-registered potential donors.

## Introduction

As of July 1, 2019, there were 113,312 candidates for an organ transplantation on waiting lists in the United States. The time on waiting lists can be quite long and varies by organ type. For example, in 2017, only 47 percent of kidney transplant candidates first listed in 2007 had received a transplant [1].

The need for more organ donations is widely recognized and many different types of efforts have been used to increase the pool of available organs. For example, donor registration efforts attempt to increase awareness of the need for organ donors and to expand the pool of individuals who have authorized organ donation [2–6]. Laws such as the state-level Uniform Anatomical Gift Act provide clarity and speed in the processing of available organs and set up guidelines for maintaining databases of registered donors [4, 7]. Training of hospital staff in protocols for obtaining authorization from family at time of death are also employed [8–12]. Authorization is not certain even for registered potential donors because of family objections [13]. Efforts to encourage and facilitate live donors for kidneys and livers have also received significant attention [14].

With this paper, we focus on the measurement of the efficiency of the donor registration process. Donor registration is the most visible method for increasing organ donation in the U.S. But there are large differences across states in registration rates, offering the possibility that perhaps some states have significant room for improvement. Donor registration drives are also a costly endeavor. Using data from Pennsylvania, researchers estimated that promotional and outreach efforts cost $455 per new registrant [15]. But the goal is to increase the number of authorized donors, not just the number of registered donors. Without detailed estimates of the cost of an additional registrant, we focus on the efficient allocation of fixed resources of time and money for increasing the number of donors.

Importantly, it is also unclear how donor registration actually links to donated organs, with increasing actual donations and transplants being the fundamental policy objective. Related to this point, some research suggests that registered donors have a lower death rate than unregistered individuals and are therefore less likely to become a potential donor [3].

In this paper we develop a new method to estimate the value of new donor registrations on organ donations. Though we use data from the United States, our method can be adapted for any country. Specifically, we measure value as the probability of a new donor registration leading to a new organ transplant donor. We use a simple model of organ donation that separates the probability of donating into its component conditional probabilities. The key probabilities include 1) the probability of becoming a potential donor, and 2) the probability of donor authorization conditional on becoming a potential donor. Using standard probability theory measures that can be linked to observable data, we create a measure of the value of a new donor registrant. This measure is then computed for various subpopulations, including state, age and gender. These statistics based on observable population-level data can then be used as a guide for directing donor registration efforts and funds toward groups or regions of highest potential value. We perform policy simulations to compare the value of different targeting strategies. We find that targeting certain states using our measure of value raises 26.7 percent more donors than a uniform, nationwide registration effort, while targeting those states with low registration rates is actually less effective than a uniform approach. We also use the estimates to show that a program to raise donor authorization rates among the non-registered population is a promising alternative.

## Methods

We use the Standard Transplant Analysis and Research (STAR) files provided by the United Network for Organ Sharing (UNOS) as contractor for the Organ Procurement and Transplantation Network (OPTN). All computations are based on OPTN data as of March 31, 2019 [16]. The “Eligible Deaths” file lists all deaths identified by Organ Procurement Organizations (OPO) due to neurological criteria (“brain dead”) as potentially eligible organ donors. The “Deceased Donor” file tracks the disposition of all donors from whom at least one usable organ was recovered irrespective of whether it was ultimately transplanted. This file includes donors identified in the Eligible Death file plus other donors who did not meet the Eligible Death criteria, but who still had available organs. For donor registration rates we use data on registration counts from Donate Life America [2]. The number of registered donors is imprecise because it is difficult to track registrants as they move between states. For our analysis, registration percentages are computed as the number of registered donors in a given group divided by the total population of that group because we consider all who might become donors. Since registration rates are not listed for demographic groups (age and gender), we supplement using estimates of these rates [17]. Population estimates used in Tables 1 and 2 come from the U.S. Census Bureau Population Division, Annual Estimates of the Resident Population 2018 [18]. Population estimates for Table 3 come from U.S. Census Bureau Population Division, Annual Estimates of the Resident Population for Selected Age Groups by Sex 2018 [19].

**Table 1:** 2018 state estimates, part 1.

State	Δ	95% CI	*P*(*R*)	*p*_1_	*p*_2_	*q*_1_	*q*_2_
All States	2.06	(1.99, 2.13)	0.46	3.81	0.96	5.14	0.56
Delaware	6.95	(3.69, 10.20)	0.61	10.21	0.98	18.41	0.60
Missouri	5.31	(4.39, 6.23)	0.59	5.19	0.98	9.90	0.45
DC	4.02	(2.38, 5.67)	0.41	3.54	1.00	6.12	0.34
Rhode Island	3.82	(2.19, 5.46)	0.48	1.77	1.00	4.92	0.22
Montana	3.62	(1.38, 5.86)	0.74	2.67	1.00	8.32	0.57
South Carolina	3.56	(2.81, 4.32)	0.42	5.08	0.94	7.08	0.43
Alaska	3.19	(0.49, 5.89)	0.71	3.43	0.94	10.35	0.64
Washington	3.06	(2.31, 3.81)	0.68	2.60	0.96	6.95	0.52
Michigan	3.02	(2.46, 3.57)	0.52	3.52	0.95	7.40	0.54
Alabama	2.97	(2.21, 3.74)	0.58	3.68	0.98	6.74	0.54
Maryland	2.96	(2.30, 3.62)	0.55	3.73	0.99	6.13	0.51
Arizona	2.81	(2.21, 3.41)	0.49	4.22	0.95	6.84	0.54
Connecticut	2.79	(2.10, 3.49)	0.38	3.77	1.00	4.82	0.42
South Dakota	2.76	(1.20, 4.33)	0.51	2.90	1.00	4.14	0.33
Hawaii	2.75	(1.40, 4.10)	0.49	3.70	0.92	5.85	0.45
Louisiana	2.65	(1.91, 3.38)	0.56	4.33	0.97	6.73	0.58
Tennessee	2.44	(1.88, 2.99)	0.35	4.66	0.94	6.98	0.60
Nevada	2.40	(1.62, 3.17)	0.45	5.23	0.99	7.45	0.66
Florida	2.40	(2.08, 2.71)	0.49	4.04	0.95	5.45	0.51
Illinois	2.36	(1.95, 2.76)	0.47	4.96	0.93	5.87	0.53
Texas	2.31	(2.06, 2.57)	0.39	3.68	0.95	5.50	0.53
Kansas	2.22	(1.31, 3.13)	0.58	4.70	0.94	6.02	0.57
New York	2.20	(1.95, 2.45)	0.28	2.93	0.99	4.46	0.50
Virginia	2.15	(1.69, 2.60)	0.53	2.90	0.99	4.73	0.54
Ohio	2.13	(1.75, 2.52)	0.49	5.21	0.96	4.63	0.50
Georgia	2.08	(1.69, 2.47)	0.46	2.69	0.98	5.18	0.58

Δ = *q*_1_(*p*_2_ − *q*_2_)

*P*(*R*) is the donor registration rate.

*p*_1_ is Potential Donor Death Rates for Registered.

*q*_1_ is Potential Donor Death Rates for Non-Registered.

*p*_2_ is Donor Authorization Rates for Registered.

*q*_2_ is Donor Authorization Rates for Non-Registered.

Entries for *p*_2_, *q*_2_ and Δ are scaled to be rates per 100,000 population.

Computations based on OPTN data as of March 31, 2019.

**Table 2:** 2018 state estimates, part 2.

State	Δ	95% CI	*P*(*R*)	*p*_1_	*p*_2_	*q*_1_	*q*_2_
All States	2.06	(1.99, 2.13)	0.46	3.81	0.96	5.14	0.56
Colorado	2.04	(1.23, 2.85)	0.77	2.54	0.94	4.93	0.52
Mississippi	1.96	(1.37, 2.55)	0.28	2.72	1.00	5.65	0.65
Pennsylvania	1.93	(1.60, 2.27)	0.38	6.70	0.96	6.06	0.64
Wisconsin	1.89	(1.39, 2.39)	0.50	4.52	1.00	4.60	0.59
New Mexico	1.78	(0.95, 2.60)	0.47	3.43	0.97	4.16	0.54
Oklahoma	1.69	(0.95, 2.42)	0.46	4.77	0.92	7.43	0.69
North Carolina	1.68	(1.27, 2.09)	0.50	3.96	0.89	4.70	0.53
West Virginia	1.68	(0.94, 2.42)	0.34	4.72	1.00	4.62	0.64
Massachusetts	1.67	(1.23, 2.12)	0.49	3.44	0.97	4.22	0.58
California	1.60	(1.42, 1.77)	0.38	3.31	0.96	3.94	0.56
Minnesota	1.56	(1.09, 2.03)	0.50	3.52	0.98	3.29	0.51
Nebraska	1.55	(0.76, 2.33)	0.42	3.98	0.97	4.53	0.63
New Jersey	1.24	(0.94, 1.54)	0.31	3.47	0.98	3.60	0.63
Arkansas	1.22	(0.67, 1.77)	0.48	2.88	1.00	3.54	0.65
Indiana	1.20	(0.74, 1.66)	0.58	3.81	0.93	4.38	0.66
Maine	1.19	(0.00, 2.38)	0.56	2.79	0.90	5.47	0.69
Iowa	0.97	(0.44, 1.49)	0.57	3.03	1.00	3.28	0.70
Wyoming	0.96	(-0.37, 2.29)	0.64	2.16	1.00	5.29	0.82
Oregon	0.93	(0.44, 1.41)	0.61	2.15	0.98	3.01	0.67
New Hampshire	0.92	(0.11, 1.73)	0.55	4.01	0.90	2.30	0.50
Utah	0.90	(0.41, 1.39)	0.54	3.19	1.00	3.96	0.77
Vermont	0.82	(-0.68, 2.32)	0.50	2.85	0.78	3.54	0.55
Puerto Rico	0.67	(0.35, 0.99)	0.21	2.42	1.00	3.83	0.82
Kentucky	0.67	(0.27, 1.06)	0.44	3.65	0.96	4.17	0.80
Idaho	0.62	(0.06, 1.17)	0.46	4.66	0.95	2.45	0.70
North Dakota	0.32	(-1.14, 1.78)	0.50	2.36	0.56	3.43	0.46

Δ = *q*_1_(*p*_2_ − *q*_2_)

*P*(*R*) is the donor registration rate.

*p*_1_ is Potential Donor Death Rates for Registered.

*q*_1_ is Potential Donor Death Rates for Non-Registered.

*p*_2_ is Donor Authorization Rates for Registered.

*q*_2_ is Donor Authorization Rates for Non-Registered.

Entries for *p*_2_, *q*_2_ and Δ are scaled to be rates per 100,000 population.

Computations based on OPTN data as of March 31, 2019.

**Table 3:** 2018 estimates, gender and age.

	Δ	95% CI	*P*(*R*)	*p*_1_	*p*_2_	*q*_1_	*q*_2_
Gender							
Female	2.00	(1.89, 2.11)	0.51	2.89	0.96	4.48	0.51
Male	2.40	(2.29, 2.51)	0.46	4.42	0.96	6.53	0.59
Age Group							
16-17	0.79	(0.52, 1.06)	0.31	2.64	0.97	3.32	0.73
18-29	1.72	(1.56, 1.88)	0.48	4.79	0.98	5.31	0.65
30-54	2.98	(2.82, 3.13)	0.51	5.55	0.97	7.46	0.57
55-74	3.40	(3.19, 3.61)	0.50	3.95	0.93	7.06	0.45

Δ = *q*_1_(*p*_2_ − *q*_2_)

*P*(*R*) is the donor registration rate.

*p*_1_ is Potential Donor Death Rates for Registered.

*q*_1_ is Potential Donor Death Rates for Non-Registered.

*p*_2_ is Donor Authorization Rates for Registered.

*q*_2_ is Donor Authorization Rates for Non-Registered.

Entries for *p*_2_, *q*_2_ and Δ are scaled to be rates per 100,000 population.

Computations based on OPTN data as of March 31, 2019.

### Donation probabilities by registration status

An organ donation occurs when Authorization is granted for organs to be recovered and transplanted from a potential deceased donor. OPTN/UNOS uses the term “Eligible Death” to refer to a deceased donor declared to be legally dead based on neurological criteria (“brain dead”) along with other specified criteria [20]. OPOs are evaluated based on the eligible death conversion rate (the number of donors per eligible death), but this practice has been questioned because eligible death status is determined by OPOs [21–25]. Many organ donations come from donors not meeting the OPTN/UNOS criteria for Eligible Death (e.g., circulatory system deaths or those over 75 years of age). We account for all potential donors in our analysis.

**Fig 1:**
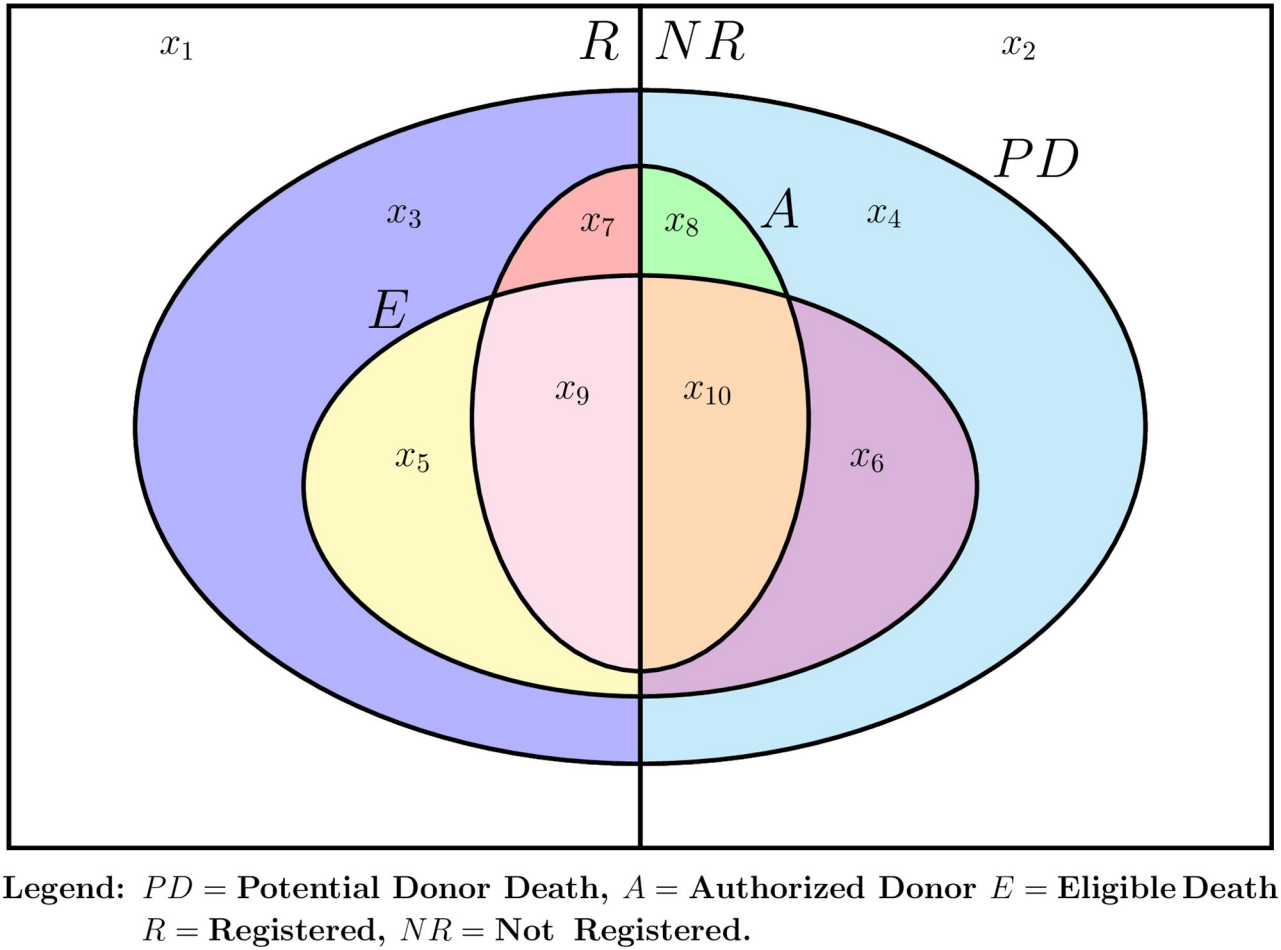
Potential donor deaths, eligible deaths, donor authorization and donor registration.

We use *R* and *NR* to denote Registered and Non-Registered donor status. Authorization for transplantation is *A*. Let *E* denote Eligible Death. Fig 1 diagrams the intersections of the various events. The vertical line divides the total population into Registered and Non-Registered groups. The outside oval is made up of all Potential Donors, *PD*, including those deficient on some element required for Eligible Death status as well as donors after circulatory death. The two inner ovals are Eligible Deaths, *E*, and Authorized Donors, *A*. We know the number of Eligible Deaths and the number of Authorized Donors from the Eligible Death and Deceased Donor files from OPTN.

The probability of donating is broken down as follows:

$$\begin{array}{ccc} P(Donate) & = & \underset{p_{2}}{\underset{︸}{P(A|PD\cap R)}}\underset{p_{1}}{\underset{︸}{P(PD|R)}}P\left(R\right)\\ & & +\underset{q_{2}}{\underset{︸}{P(A|PD\cap NR)}}\underset{q_{1}}{\underset{︸}{P(PD|NR)}}P\left(NR\right)\\ & = & p_{2}p_{1}P\left(R\right)+q_{2}q_{1}P\left(NR\right) \end{array}$$

with the conditional probabilities *p*_*i*_ and *q*_*i*_ defined as

$$\begin{array}{ccc} p_{1} & = & Pr(Potential\;Donor|Registered) \end{array}$$ (1)

$$\begin{array}{ccc} p_{2} & = & Pr(Authorized|Potential\;\;Donor\;\;and\;\;Registered) \end{array}$$ (2)

$$\begin{array}{ccc} q_{1} & = & Pr(Potential\;Donor|Not\;Registered) \end{array}$$ (3)

$$\begin{array}{ccc} q_{2} & = & Pr(Authorized|Potential\;\;Donor\;\;and\;\;Not\;\;Registered). \end{array}$$ (4)

Breaking the overall probability of donating into component conditional probabilities allows us to estimate these separately and better understand variation in donation probability across states and demographic groups. We can then simulate the effects of changes in the parameters.

### Modeling policy interventions

Next we model the effect of policy interventions on the total number of organs donated. Assume people in a population of size *M* are either Registered (*R*) or Non-Registered (*NR*). If there is homogeneity within each group, total (expected) donors, *T*, can be written as

$$\begin{array}{c} T=Rp_{1}p_{2}+\left(M-R\right)q_{1}q_{2}. \end{array}$$ (5)

A simple way to model the change would be to assume that donor registration increases the size of the registered population and decreases the size of the non-registered population. That is, a new donor registration increases *R* by one and decreases *NR* by one, yielding a net change in total expected donors of *p*_1_ *p*_2_ − *q*_1_ *q*_2_. The change will be larger when there is a large difference in the conditional authorization rates (*p*_2_ and *q*_2_), or when the death rates (*p*_1_ and *q*_1_) differ substantially between registered and non-registered populations.

While this modeling of the effect of registration on *T* accounts for the offsetting reduction from the unregistered, it implicitly assumes that registration converts the registrant from one type to the other, changing not just the conditional probability of Donor Authorization but also the probability of becoming a Potential Donor. A more realistic assumption is that registration changes only the conditional Authorization rate *p*_2_, leaving the other parameters unchanged. Then the expected increase in the number of donors from a new registration is

$$\begin{array}{c} \Delta =q_{1}p_{2}-q_{1}q_{2}=q_{1}\left(p_{2}-q_{2}\right) \end{array}$$ (6)

We can then compute Δ by state and for various demographic subgroups. Eq (6) accounts for the fact that the value of a registration is lower when families of non-registrants are likely to authorize donation.

Estimation of Δ is straightforward given suitable data: simply estimate the conditional probabilities (1)–(4). We deal with two difficulties related to missing or incomplete data. First, both the Eligible Death file and the Deceased Donor file record registration status, but they do not always agree. Given that the Deceased Donors file is the last record of registration status, we use that measure of registration for all donors in that file. For the set of potential donors in the Eligible Death file who do not donate, we use the registration status from the Eligible Death file. Eligible Deaths who donate organs are also listed in the Deceased Donor file, so for this set we have both measures. We find that the two measures agree for 7,882 of 8,272 such observations, with a correlation coefficient of 0.91.

The second difficulty is that we do not know the number of Potential Donors. Potential Donors is a smaller set than the total number of deaths. It is the set of individuals who meet the medical criteria for having at least one donatable organ upon death. In practice this number is not collected or reported for the whole population.

However, we can estimate it based on the data from Eligible Deaths. The set of Eligible Deaths is well defined on neurological criteria and a set of other restrictions. From these data we can estimate the Authorization rate for Eligible Deaths. If we make the plausible assumption that the Authorization rate is the same for *all* deaths (e.g. including deaths from circulatory system failure), then we can estimate the number of registered and non-registered Potential Donors based on the observed number of actual Authorized donors from each group. We also account for the statistical uncertainty of our estimates and assumptions. Specific details for the computations are found in the Appendix.

Ideally, we would also like to compute the value of a new registration, Δ, for demographic characteristics such as age, gender, ethnicity, and religious affiliation. However, state-level data for donor registries with demographic details are not generally available. Unlike donor registries in other countries, no information is collected on the religious affiliation of deceased organ donors or new donor registrants in the United States. Likewise, at the most common location to register as a organ donor in the United States, each state’s Department of Motor Vehicles, no information on ethnicity is recorded. We do have demographic data on age and gender for a subset of 12 states (AZ, CA, FL, GA, ID, OK, OR, SC, TX, UT, VA, WA) plus Puerto Rico and the District of Columbia from a 2015 study commissioned by Donate Life America (DLA). We use these estimates to do a more detailed analysis on the available subset of states [17, 26].

## Results

**Fig 2:**
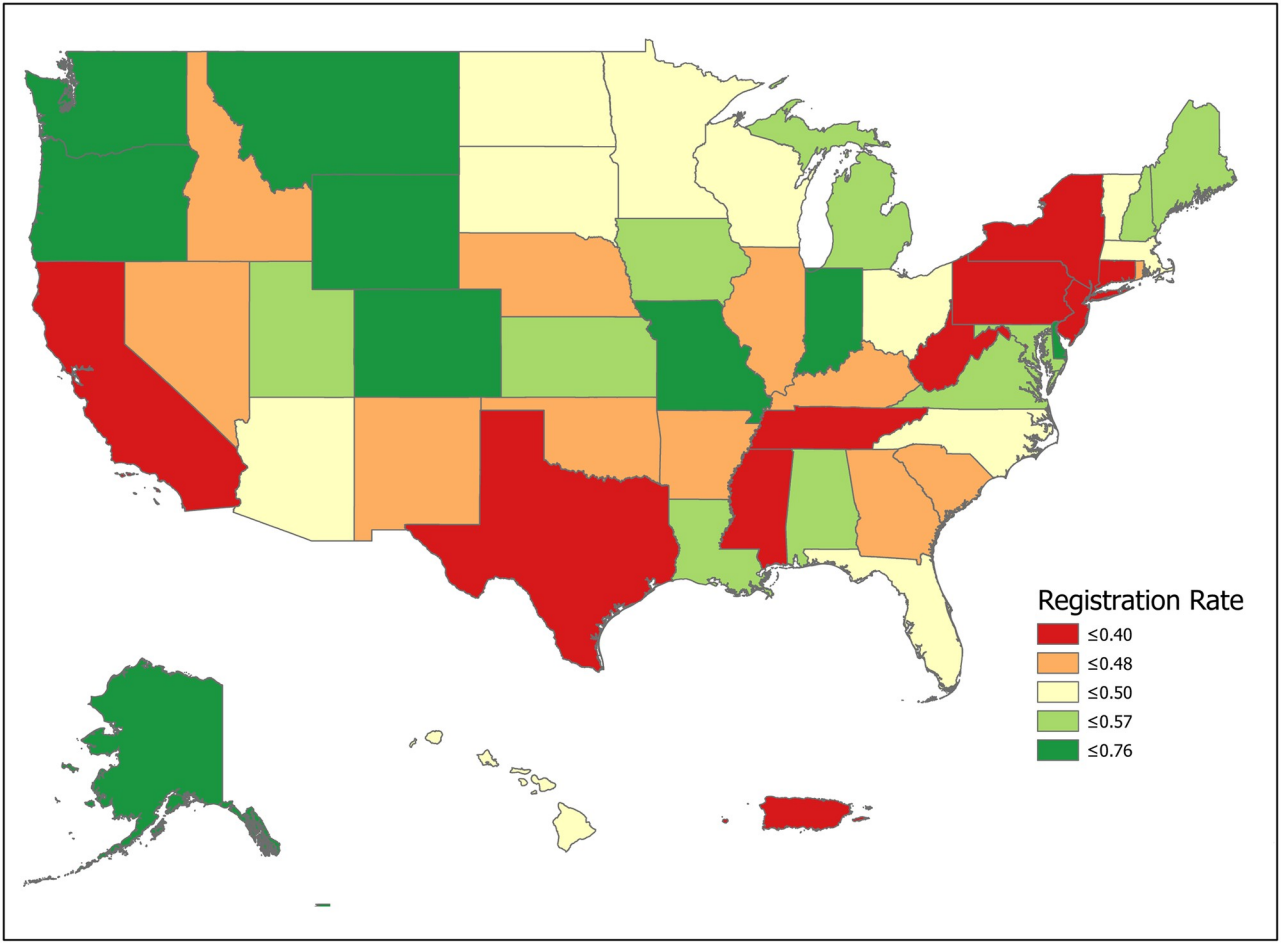
2018 state donor registration rates. Data come from Tables 1 and 2. Maps created by ArcGIS Pro, URL: https://www.esri.com/en-us/arcgis/products/arcgis-pro/overview. Base Map: World Light Gray Base, URL: https://services.arcgisonline.com/arcgis/rest/services/Canvas.

**Fig 3:**
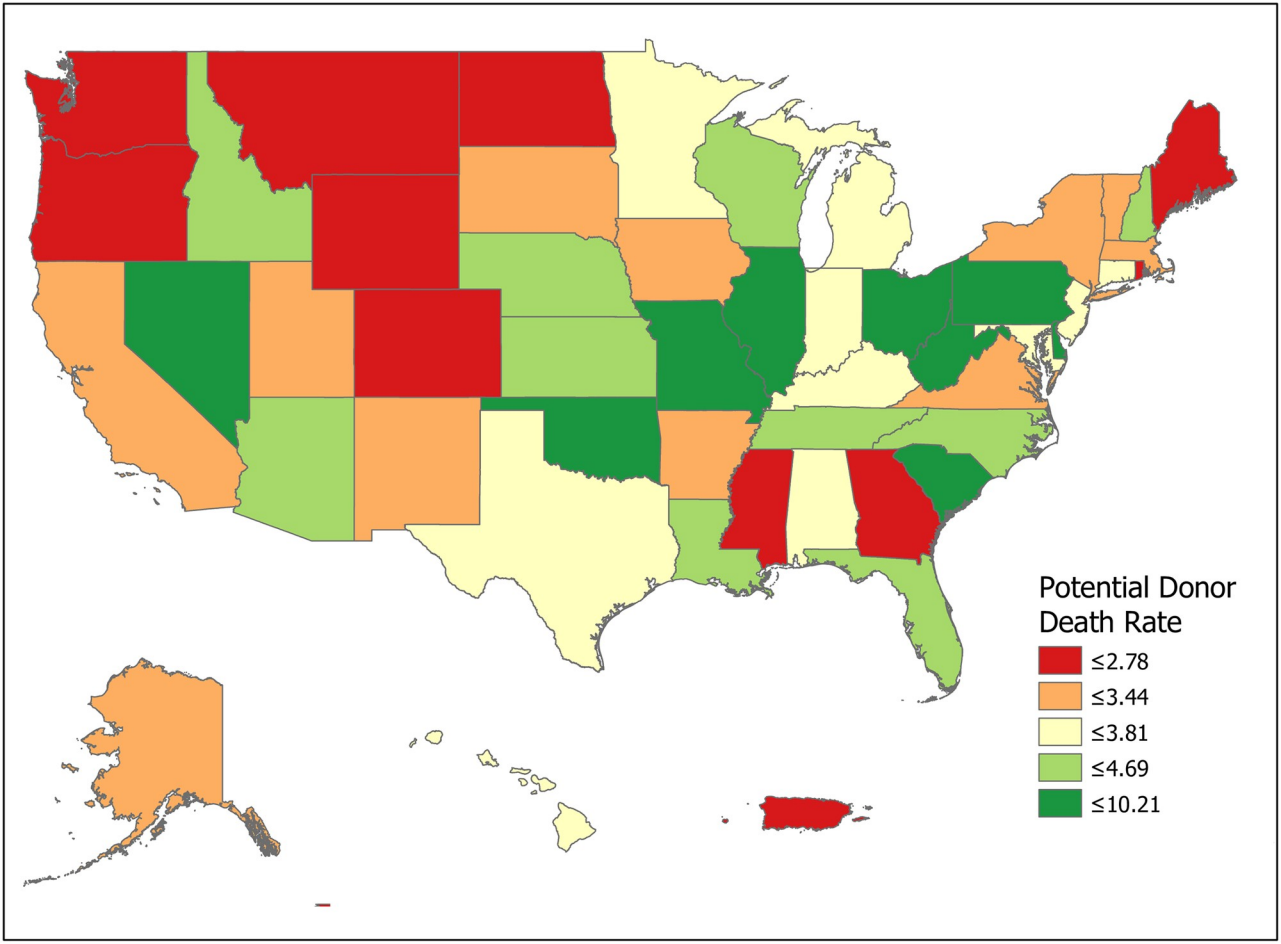
2018 potential donor death rates, registered (*p*_1_). Data come from Tables 1 and 2. Maps created by ArcGIS Pro, URL: https://www.esri.com/en-us/arcgis/products/arcgis-pro/overview. Base Map: World Light Gray Base, URL: https://services.arcgisonline.com/arcgis/rest/services/Canvas.

**Fig 4:**
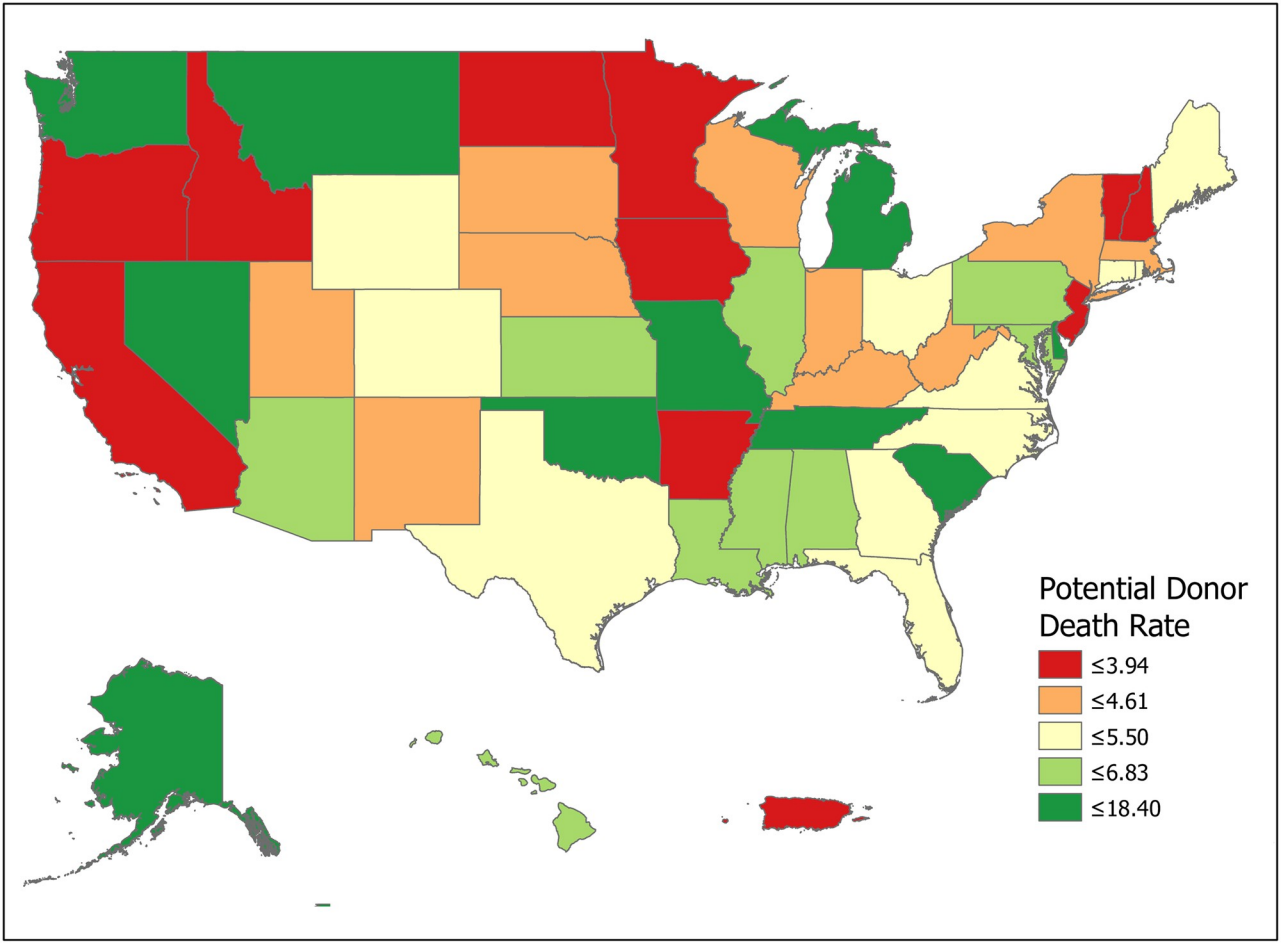
2018 potential donor death rates, non-registered (*q*_1_). Data come from Tables 1 and 2. Maps created by ArcGIS Pro, URL: https://www.esri.com/en-us/arcgis/products/arcgis-pro/overview. Base Map: World Light Gray Base, URL: https://services.arcgisonline.com/arcgis/rest/services/Canvas.

**Fig 5:**
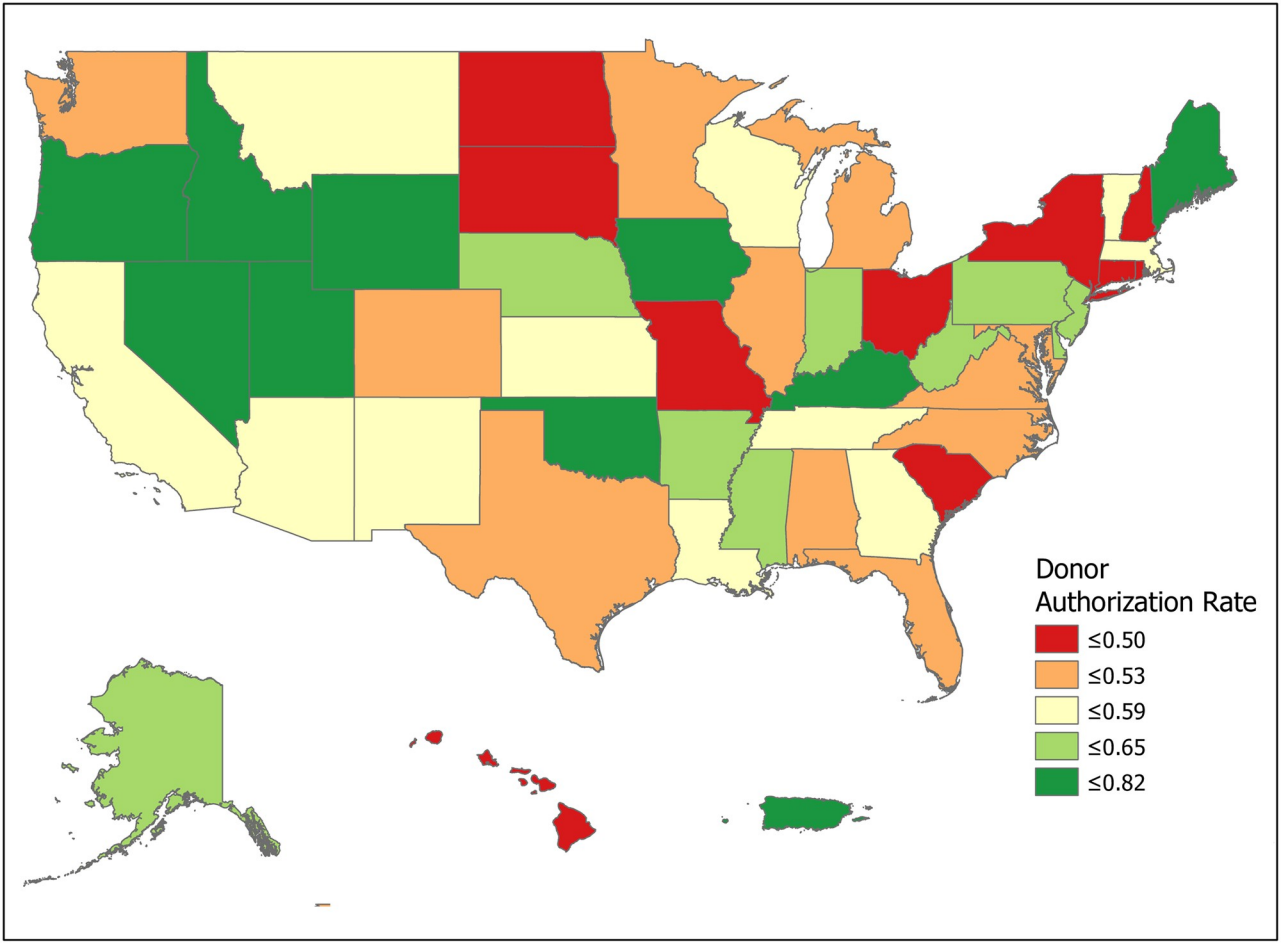
2018 donor authorization rates, non-registered (*q*_2_). Data come from Tables 1 and 2. Maps created by ArcGIS Pro, URL: https://www.esri.com/en-us/arcgis/products/arcgis-pro/overview. Base Map: World Light Gray Base, URL: https://services.arcgisonline.com/arcgis/rest/services/Canvas.

We can compute the value of a new registration, Δ, using Eq (6) for various subpopulations including for all states and for the demographic subgroups for which we have data. Tables 1 and 2 compute Δ for 50 states plus the District of Columbia and Puerto Rico. Confidence intervals for *p*_1_ (Registered Potential Donor Death rate), *p*_2_ (Registered Donor Authorization rate), *q*_1_ (Non-Registered Potential Donor Death rate) and *q*_2_ (Non-Registered Donor Authorization rate) for each state are found in Tables 4 and 5 in the Appendix. Note that we have scaled estimates for *p*_1_, *q*_1_ and Δ to rates per 100,000 population. Figs 2–5 show the variation across states of Registration rates, Potential Donor Donor Death rates by registration status, and the Non-Registered Donor Authorization rate. These figures were created from our computations using ESRI ARCGIS mapping software [27].

**Table 4:** Confidence intervals for *p*_1_, *p*_2_, *q*_1_, and *q*_2_, Alaska-Mississippi.

State	*p*_1_	95% CI	*p*_2_	95% CI	*q*_1_	95% CI	*q*_2_	95% CI
U.S.	3.81	(3.71, 3.91)	0.96	(0.95, 0.96)	5.14	(5.04, 5.25)	0.56	(0.55, 0.57)
AK	3.43	(1.84, 5.01)	0.94	(0.84, 1.05)	10.35	(6.03, 14.68)	0.64	(0.44, 0.84)
AL	3.68	(2.97, 4.39)	0.98	(0.95, 1.01)	6.74	(5.62, 7.86)	0.54	(0.46, 0.62)
AR	2.88	(2.01, 3.74)	1.00	(1.00, 1.00)	3.54	(2.61, 4.48)	0.65	(0.53, 0.78)
AZ	4.22	(3.54, 4.90)	0.95	(0.92, 0.99)	6.84	(5.99, 7.69)	0.54	(0.48, 0.60)
CA	3.31	(3.02, 3.60)	0.96	(0.94, 0.98)	3.94	(3.70, 4.19)	0.56	(0.52, 0.59)
CO	2.54	(2.06, 3.01)	0.94	(0.89, 0.98)	4.93	(3.73, 6.13)	0.52	(0.40, 0.64)
CT	3.77	(2.73, 4.80)	1.00	(1.00, 1.00)	4.82	(3.91, 5.74)	0.42	(0.33, 0.51)
DC	3.54	(1.69, 5.40)	1.00	(1.00, 1.00)	6.12	(4.09, 8.15)	0.34	(0.19, 0.50)
DE	10.21	(7.20, 13.23)	0.98	(0.93, 1.02)	18.41	(13.31, 23.51)	0.60	(0.46, 0.74)
FL	4.04	(3.65, 4.42)	0.95	(0.93, 0.97)	5.45	(5.01, 5.89)	0.51	(0.47, 0.55)
GA	2.69	(2.23, 3.16)	0.98	(0.96, 1.01)	5.18	(4.59, 5.77)	0.58	(0.53, 0.64)
HI	3.70	(2.28, 5.13)	0.92	(0.82, 1.03)	5.85	(4.08, 7.61)	0.45	(0.30, 0.60)
IA	3.03	(2.23, 3.84)	1.00	(1.00, 1.00)	3.28	(2.31, 4.24)	0.70	(0.57, 0.84)
ID	4.66	(3.18, 6.15)	0.95	(0.88, 1.02)	2.45	(1.45, 3.45)	0.70	(0.51, 0.88)
IL	4.96	(4.39, 5.53)	0.93	(0.90, 0.96)	5.87	(5.29, 6.44)	0.53	(0.48, 0.58)
IN	3.81	(3.20, 4.43)	0.93	(0.89, 0.97)	4.38	(3.60, 5.15)	0.66	(0.57, 0.74)
KS	4.70	(3.66, 5.73)	0.94	(0.88, 0.99)	6.02	(4.65, 7.39)	0.57	(0.45, 0.68)
KY	3.65	(2.81, 4.49)	0.96	(0.91, 1.00)	4.17	(3.37, 4.97)	0.80	(0.72, 0.88)
LA	4.33	(3.53, 5.13)	0.97	(0.94, 1.00)	6.73	(5.60, 7.85)	0.58	(0.50, 0.66)
MA	3.44	(2.82, 4.07)	0.97	(0.95, 1.00)	4.22	(3.54, 4.89)	0.58	(0.50, 0.66)
MD	3.73	(3.07, 4.38)	0.99	(0.98, 1.01)	6.13	(5.20, 7.07)	0.51	(0.43, 0.59)
ME	2.79	(1.59, 3.98)	0.90	(0.78, 1.03)	5.47	(3.58, 7.37)	0.69	(0.53, 0.85)
MI	3.52	(3.01, 4.03)	0.95	(0.91, 0.98)	7.40	(6.63, 8.17)	0.54	(0.49, 0.59)
MN	3.52	(2.82, 4.21)	0.98	(0.95, 1.01)	3.29	(2.62, 3.96)	0.51	(0.40, 0.61)
MO	5.19	(4.45, 5.93)	0.98	(0.97, 1.00)	9.90	(8.67, 11.14)	0.45	(0.39, 0.51)
MS	2.72	(1.61, 3.83)	1.00	(1.00, 1.00)	5.65	(4.64, 6.66)	0.65	(0.57, 0.74)

Δ = *q*_1_(*p*_2_ − *q*_2_)

*P*(*R*) is the donor registration rate.

*p*_1_ is Potential Donor Death Rates for Registered.

*q*_1_ is Potential Donor Death Rates for Non-Registered.

*p*_2_ is Donor Authorization Rates for Registered.

*q*_2_ is Donor Authorization Rates for Non-Registered.

Entries for *p*_2_, *q*_2_ and Δ are scaled to be rates per 100,000 population.

Computations based on OPTN data as of March 31, 2019.

**Table 5:** Confidence intervals for *p*_1_, *p*_2_, *q*_1_, and *q*_2_, Montana-Wyoming.

State	*p*_1_	95% CI	*p*_2_	95% CI	*q*_1_	95% CI	*q*_2_	95% CI
U.S.	3.81	(3.71, 3.91)	0.96	(0.95, 0.96)	5.14	(5.04, 5.25)	0.56	(0.55, 0.57)
MT	2.67	(1.53, 3.81)	1.00	(1.00, 1.00)	8.32	(4.92, 11.72)	0.57	(0.36, 0.77)
NC	3.96	(3.42, 4.50)	0.89	(0.84, 0.93)	4.70	(4.11, 5.29)	0.53	(0.47, 0.59)
ND	2.36	(0.82, 3.90)	0.56	(0.23, 0.88)	3.43	(1.57, 5.29)	0.46	(0.19, 0.73)
NE	3.98	(2.60, 5.36)	0.97	(0.91, 1.03)	4.53	(3.29, 5.78)	0.63	(0.49, 0.76)
NH	4.01	(2.57, 5.44)	0.90	(0.79, 1.01)	2.30	(1.10, 3.51)	0.50	(0.24, 0.76)
NJ	3.47	(2.78, 4.16)	0.98	(0.95, 1.01)	3.60	(3.12, 4.07)	0.63	(0.57, 0.70)
NM	3.43	(2.28, 4.59)	0.97	(0.91, 1.03)	4.16	(2.96, 5.36)	0.54	(0.40, 0.69)
NV	5.23	(4.02, 6.45)	0.99	(0.96, 1.01)	7.45	(6.14, 8.76)	0.66	(0.58, 0.75)
NY	2.93	(2.48, 3.38)	0.99	(0.98, 1.01)	4.46	(4.11, 4.81)	0.50	(0.46, 0.54)
OH	5.21	(4.62, 5.80)	0.96	(0.94, 0.98)	4.63	(4.08, 5.17)	0.50	(0.44, 0.56)
OK	4.77	(3.76, 5.78)	0.92	(0.86, 0.98)	7.43	(6.28, 8.59)	0.69	(0.62, 0.76)
OR	2.15	(1.58, 2.71)	0.98	(0.95, 1.02)	3.01	(2.17, 3.85)	0.67	(0.54, 0.80)
PA	6.70	(5.97, 7.43)	0.96	(0.94, 0.98)	6.06	(5.52, 6.60)	0.64	(0.60, 0.68)
PR	2.42	(1.24, 3.61)	1.00	(1.00, 1.00)	3.83	(3.07, 4.59)	0.82	(0.75, 0.90)
RI	1.77	(0.61, 2.93)	1.00	(1.00, 1.00)	4.92	(3.06, 6.77)	0.22	(0.07, 0.38)
SC	5.08	(4.12, 6.03)	0.94	(0.89, 0.98)	7.08	(6.12, 8.04)	0.43	(0.37, 0.50)
SD	2.90	(1.32, 4.48)	1.00	(1.00, 1.00)	4.14	(2.23, 6.06)	0.33	(0.12, 0.55)
TN	4.66	(3.78, 5.53)	0.94	(0.90, 0.99)	6.98	(6.20, 7.75)	0.60	(0.54, 0.65)
TX	3.68	(3.32, 4.04)	0.95	(0.93, 0.97)	5.50	(5.16, 5.85)	0.53	(0.50, 0.56)
UT	3.19	(2.35, 4.04)	1.00	(1.00, 1.00)	3.96	(2.93, 4.99)	0.77	(0.66, 0.88)
VA	2.90	(2.40, 3.40)	0.99	(0.98, 1.01)	4.73	(4.06, 5.41)	0.54	(0.47, 0.61)
VT	2.85	(0.99, 4.71)	0.78	(0.51, 1.05)	3.54	(1.45, 5.64)	0.55	(0.25, 0.84)
WA	2.60	(2.16, 3.04)	0.96	(0.92, 0.99)	6.95	(5.89, 8.01)	0.52	(0.44, 0.59)
WI	4.52	(3.74, 5.29)	1.00	(1.00, 1.00)	4.60	(3.82, 5.38)	0.59	(0.51, 0.67)
WV	4.72	(3.00, 6.43)	1.00	(1.00, 1.00)	4.62	(3.40, 5.84)	0.64	(0.51, 0.76)
WY	2.16	(0.66, 3.66)	1.00	(1.00, 1.00)	5.29	(2.16, 8.41)	0.82	(0.59, 1.05)

Δ = *q*_1_(*p*_2_ − *q*_2_)

*P*(*R*) is the donor registration rate.

*p*_1_ is Potential Donor Death Rates for Registered.

*q*_1_ is Potential Donor Death Rates for Non-Registered.

*p*_2_ is Donor Authorization Rates for Registered.

*q*_2_ is Donor Authorization Rates for Non-Registered.

Entries for *p*_2_, *q*_2_ and Δ are scaled to be rates per 100,000 population.

Computations based on OPTN data as of March 31, 2019.

**Fig 6:**
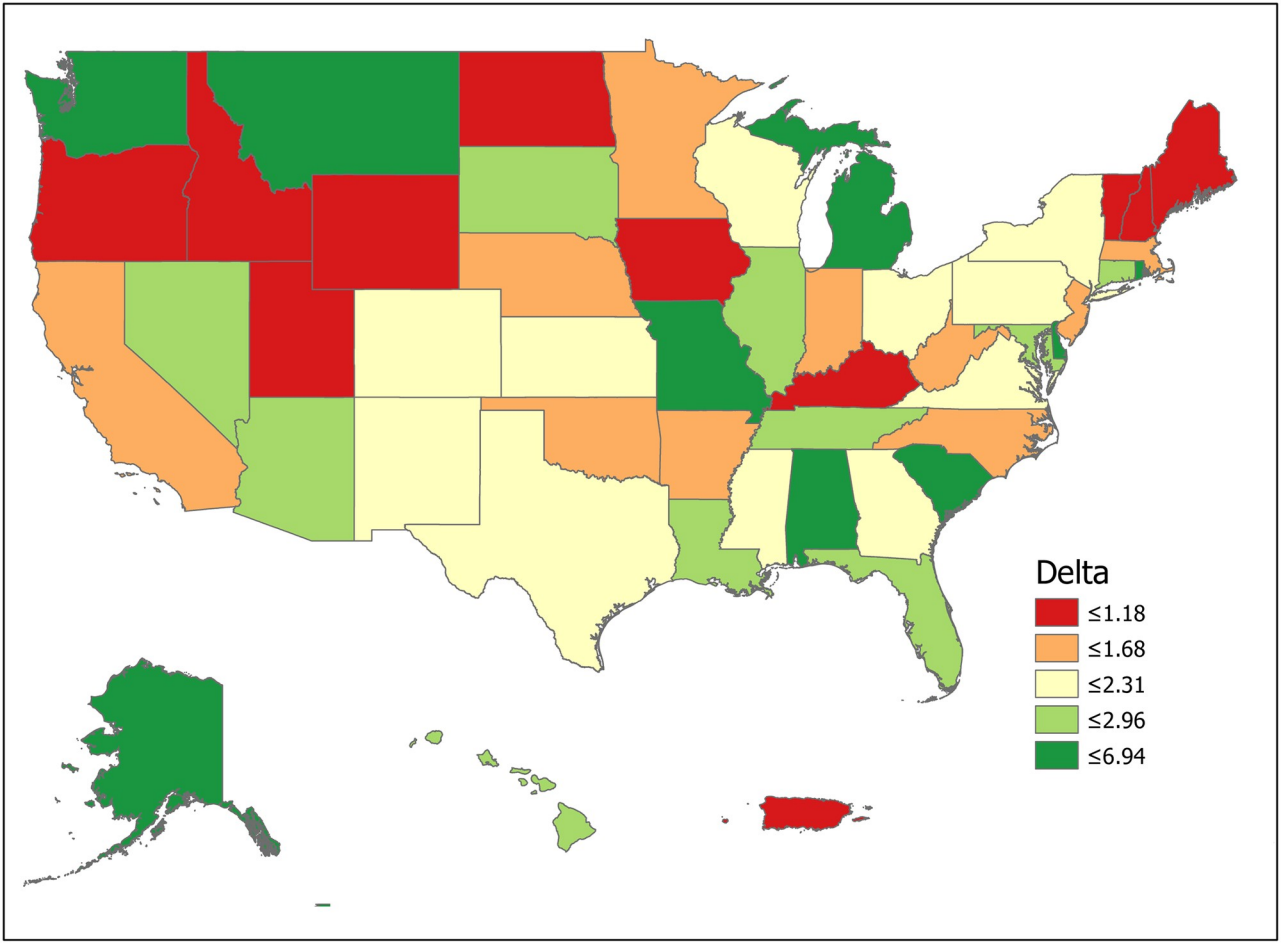
2018 estimated value of an additional registered donor (Δ). Data come from Tables 1 and 2. Maps created by ArcGIS Pro, URL: https://www.esri.com/en-us/arcgis/products/arcgis-pro/overview. Base Map: World Light Gray Base, URL: https://services.arcgisonline.com/arcgis/rest/services/Canvas.

Estimated values of Δ should be large when non-registered Potential Donor death rates are high and when non-registered Authorization rates are low. A glance at Fig 6 suggests regional correlation. For example, Δ tends to be larger in the southern states, though Arkansas and North Carolina are important exceptions.

For the U.S. as a whole, the value of Δ is 2.06 per 100,000, meaning that 100,000 new registrants should produce an increase of 2.06 donors per year. State estimates of Δ range from lows of 0.32 and 0.62 in North Dakota and Idaho to highs of 6.95 and 5.31 in Delaware and Missouri. Point estimates suggest that the relative value of a new registrant is 21.7 times higher in Delaware than in North Dakota (6.95/0.32). The ratio of the 75th percentile (South Dakota, 2.76) to the 25th percentile (Arkansas, 1.22) is 2.26. Similarly, the relative value of a new registrant in Connecticut is 67 percent higher than that of one in neighboring Massachusetts (2.79 vs. 1.67). The precision of estimation improves for more populous states. The largest states by population are California (Δ = 1.60, CI = (1.42,1.77)), Texas (2.31, CI = (2.06, 2.57)), Florida (2.40, CI = (2.08, 2.71)) and New York (2.20, CI = (1.95, 2.45)).

We now look closely at the influence of the component probabilities on the magnitude of Δ. The national donor registration rate is 46 percent and state rates range from 21 percent in Puerto Rico to 77 percent in Colorado. The four largest states (California, Texas, Florida and New York) have relatively low registration rates. The national Potential Donor Death rate (*p*_1_ or *q*_1_) is higher for the non-registered than for the registered, and this pattern holds for most states. What matters for the value of Δ is the non-registered Potential Donor Death rate *q*_1_. This ranges from a high of 18.41 in Delaware to a low of 2.30 in New Hampshire. However, high Donor Authorization rates for the non-registered (*q*_2_) reduce the effect of higher Potential Donor Death rates because the non-registered Potential Donor Deaths are already highly likely to become donors. For example, Pennsylvania’s Potential Donor Death rate rate is high (6.06) but its Δ is lower than average (1.93, CI = (1.60, 2.27)) because it has a relatively high Donor Authorization rate of 0.64 (CI = (0.60, 0.68)). In contrast, New York—with a similar Δ of 2.20—has a lower Potential Donor Death rate and Donor Authorization rate for the non-registered. These Donor Authorization rates vary from a high of 81 percent in Puerto Rico to a low of 26 percent in Vermont, while the national average rate is 56 percent.

Table 3 shows estimates by Gender and Age for the subset of states (14) for which we have data. As noted above, Donate Life America (DLA) does not record the detailed demographic information of registered donors. DLA commissioned consulting firm Bach Harrison LLC to use registrant zipcodes to estimate registration rates for these subgroups [17]. We use these registration estimates from 2015 and match them with 2018 OPTN data on deaths and donations.

Males are less likely to be registered than Females (46 vs. 51 percent), but the estimated non-registered Donor Authorization rate (*q*_2_) is slightly higher for men (62 vs 57 percent). The dominant factor here is that the non-registered Potential Donor death rate is 46 percent higher for men (6.53 vs. 4.48). Therefore, the estimated Δ for men is 2.40 and only 2.00 for women, or 20 percent higher.

Estimates for age groups show similar patterns. Registration rates are fairly constant across age groups other than the youngest group, but non-registered Donor Authorization rates (*q*_2_) fall from 65 percent for the 18-29 age group to 45 percent for the 55-74 age group. The estimated values for Δ rise markedly from 1.72 for the 18-29 group to 2.98 for ages 30-54 and 3.40 for ages 55-74. These estimates are by necessity short-run computations that ignore many complexities such as migration between states and the difference in the number of years on a donor registry for the middle-aged versus young adults. Since our estimate is the value per year, it may understate the benefit of registering younger donors.

### Policy simulations

The estimates of Δ and the probabilities in Tables 1–3 show significant variation across states and demographic groups. We now show how the estimates can be used to evaluate the effects of policy changes aimed at increasing the number of donors. Table 6 shows the results of eight policy simulations. Each effect is evaluated at the state level using Tables 1 and 2 and then aggregated to report the national effect.

**Table 6:** Policy simulations.

		Donors	Increase	Percent Increase
Baseline	Total Donors, 2018	10,670	–	–
Simulation 1	Increase *R* by 10% in each state.	10,999	329	3.08%
Simulation 2	Increase *R* 20% in High Delta States	11,087	417	3.91%
Simulation 3	Increase *R* 20% in low Registration rate States	10,969	299	2.80%
Simulation 4	Increase *R* 20% in low NR Donor Authorization (*q*_2_) States	11,054	384	3.60%
Simulation 5	Increase *R* 20% in high NR Potential Donor Death Rate (*q_1_*) States	11,076	406	3.80%
Simulation 6	Increase Non-Registered Donor Authorization Rate (*q*_2_) 5 percentage points.	11,133	463	4.34%
Simulation 7	Increase Non-Registered Donor Authorization Rate (*q*_2_) 10 percentage points.	11,596	926	8.68%
Simulation 8	Raise Non-Registered Donor Authorization Rate (*q*_2_) to 0.6.	11,211	541	5.07%

Simulations 1-5 use Δ(Change in R), state by state.

Simulations 6-8 use *T = Rp_1_p_2_ + NRq_1_q_2_* (Eq 5), changing parameters as noted in the text.

**Fig 7:**
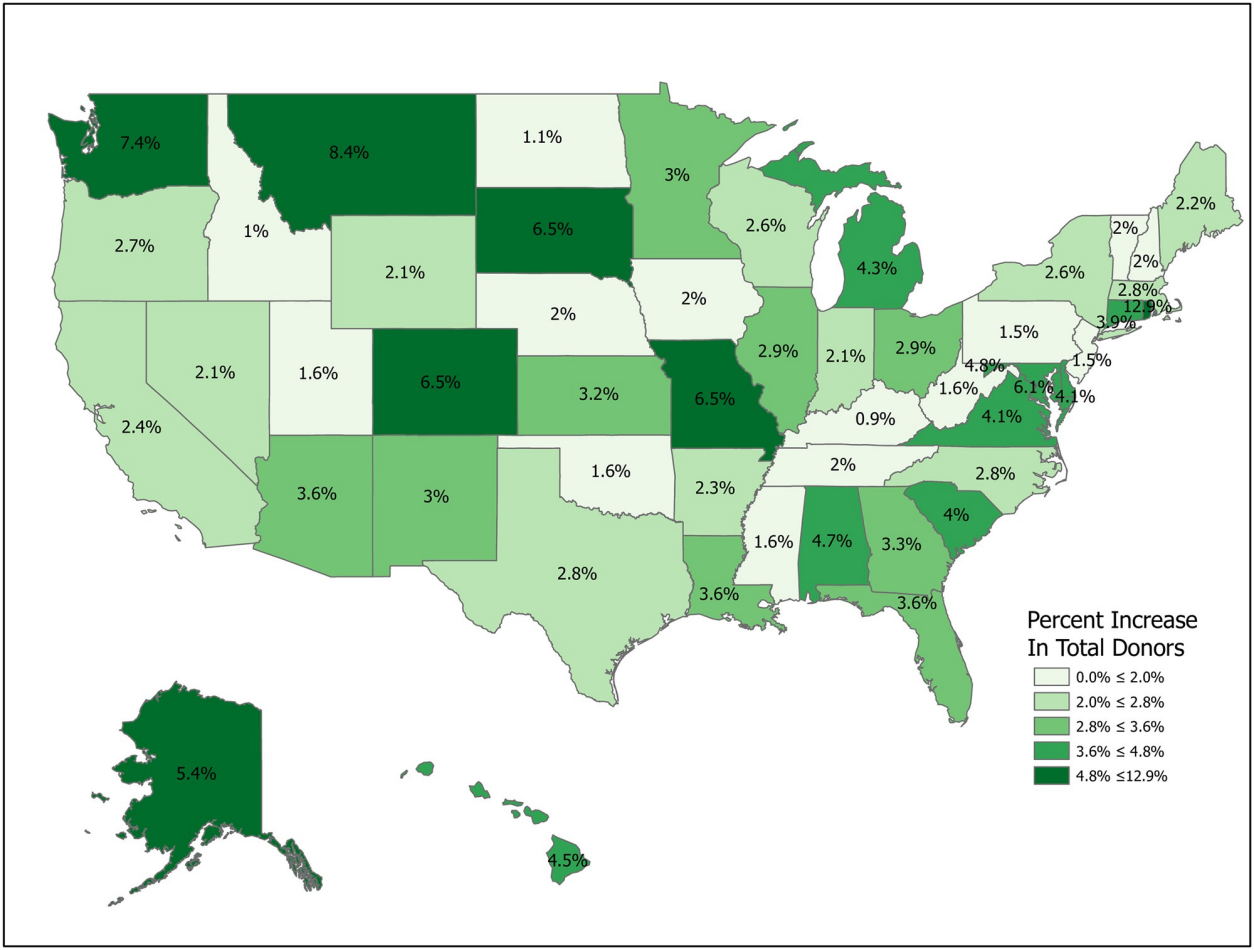
Simulation 1: Increase registration rates by 10 percent in each state. Data come from Table 6. Maps created by ArcGIS Pro, URL: https://www.esri.com/en-us/arcgis/products/arcgis-pro/overview. Base Map: World Light Gray Base, URL: https://services.arcgisonline.com/arcgis/rest/services/Canvas.

**Fig 8:**
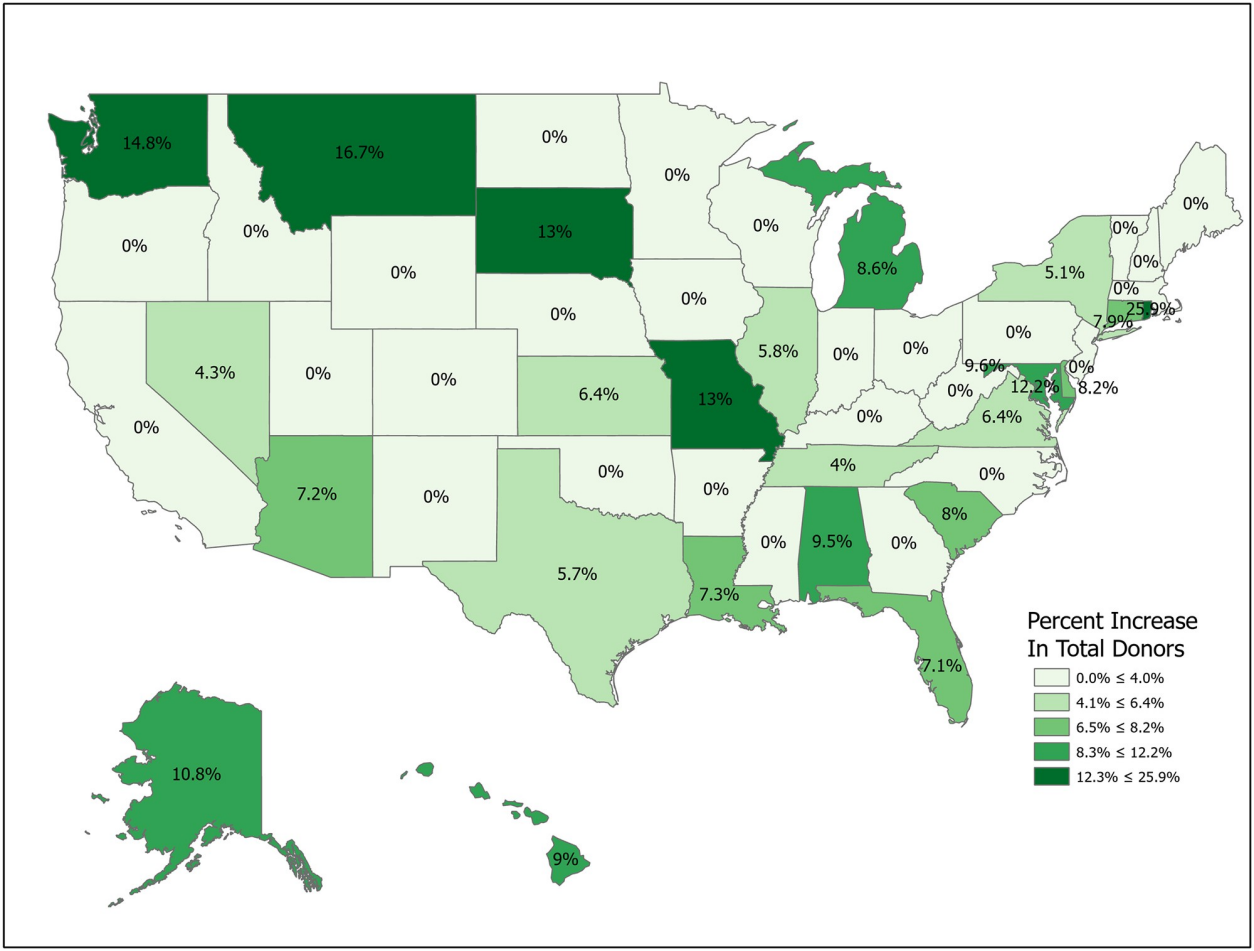
Simulation 2: Target high Δ states. Data come from Table 6. Maps created by ArcGIS Pro, URL: https://www.esri.com/en-us/arcgis/products/arcgis-pro/overview. Base Map: World Light Gray Base, URL: https://services.arcgisonline.com/arcgis/rest/services/Canvas.

**Fig 9:**
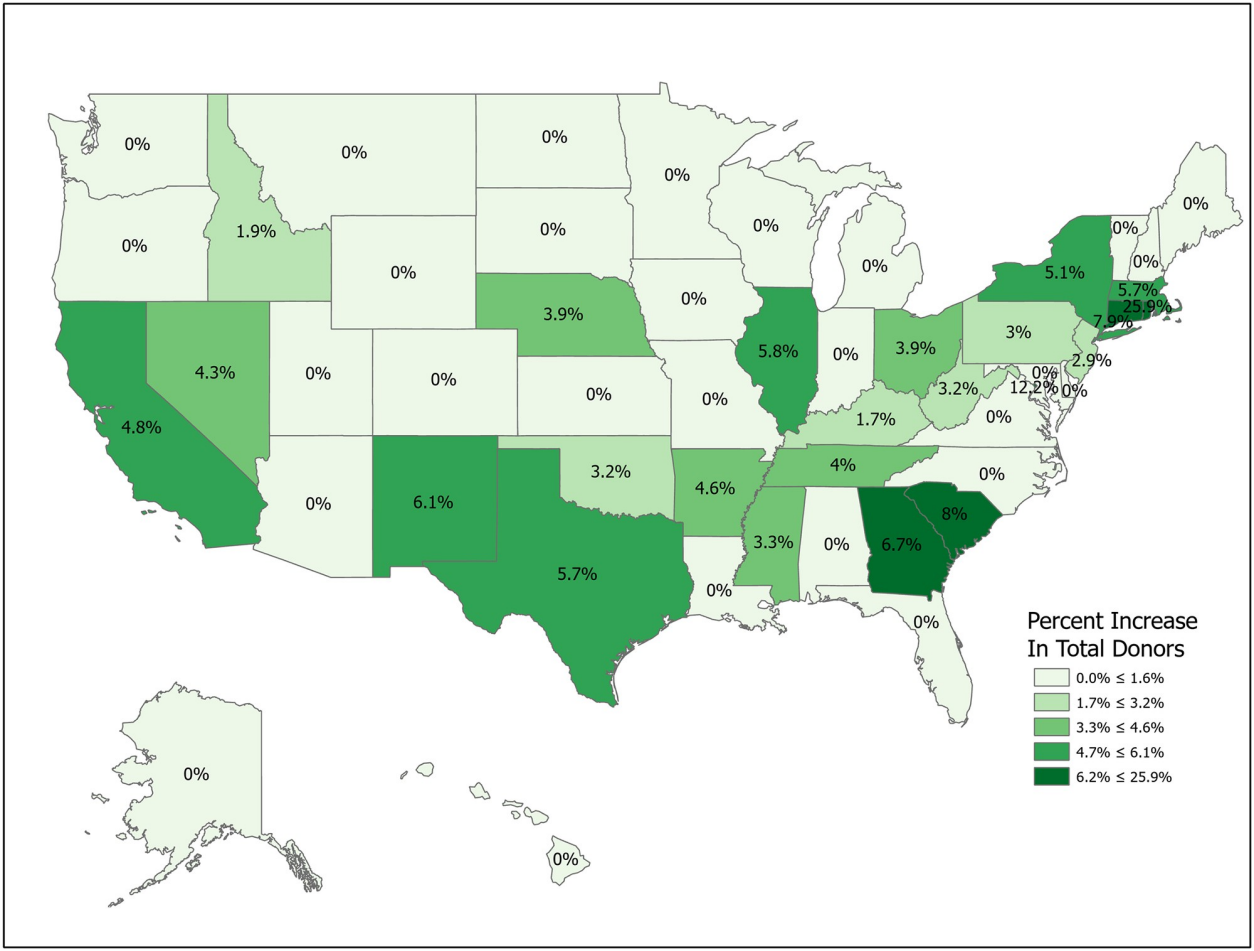
Simulation 3: Target states with low donor registration rates. Data come from Table 6. Maps created by ArcGIS Pro, URL: https://www.esri.com/en-us/arcgis/products/arcgis-pro/overview. Base Map: World Light Gray Base, URL: https://services.arcgisonline.com/arcgis/rest/services/Canvas.

**Fig 10:**
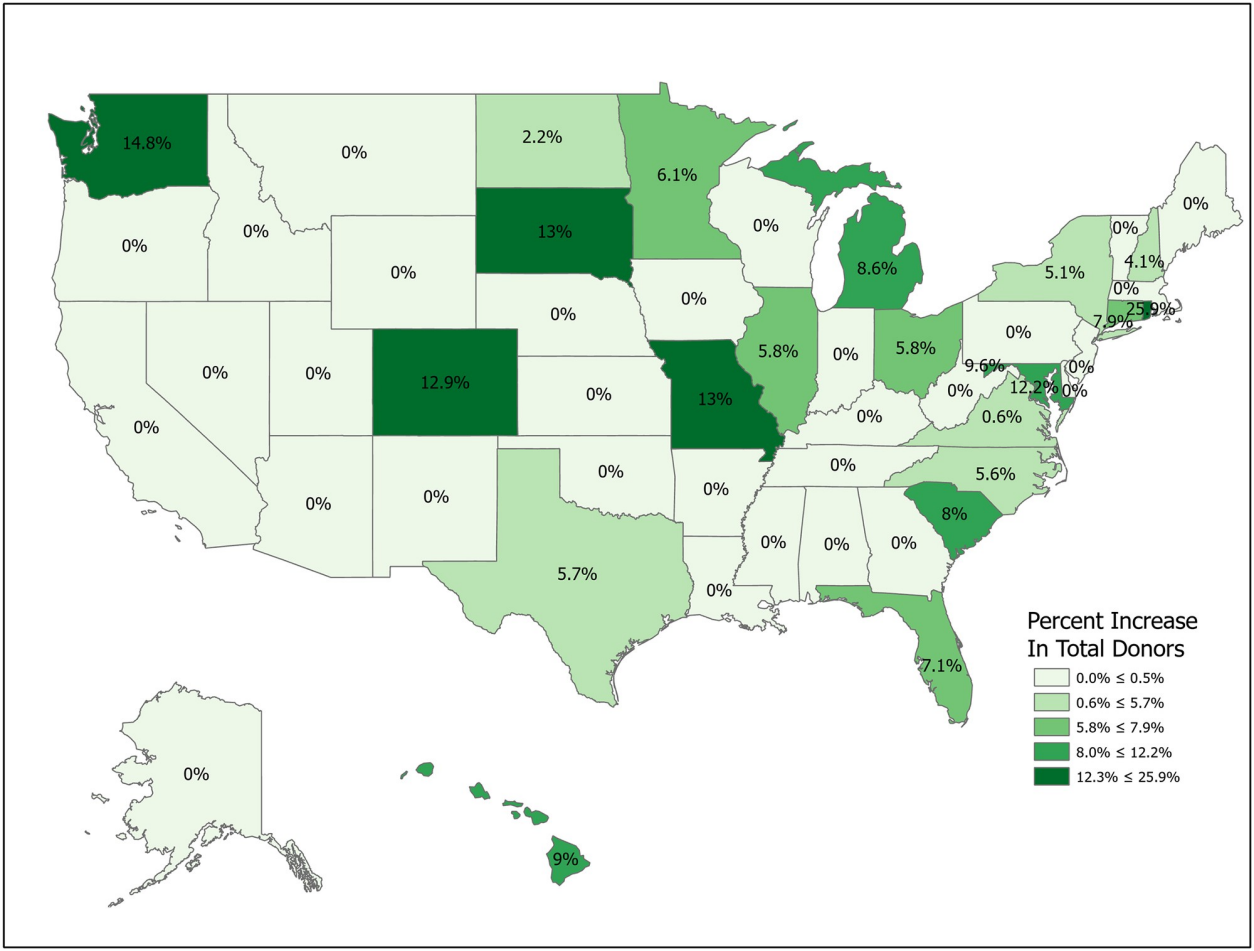
Simulation 4: Target states with low non-registered donor authorization rates (*q*_2_). Data come from Table 6. Maps created by ArcGIS Pro, URL: https://www.esri.com/en-us/arcgis/products/arcgis-pro/overview. Base Map: World Light Gray Base, URL: https://services.arcgisonline.com/arcgis/rest/services/Canvas.

**Fig 11:**
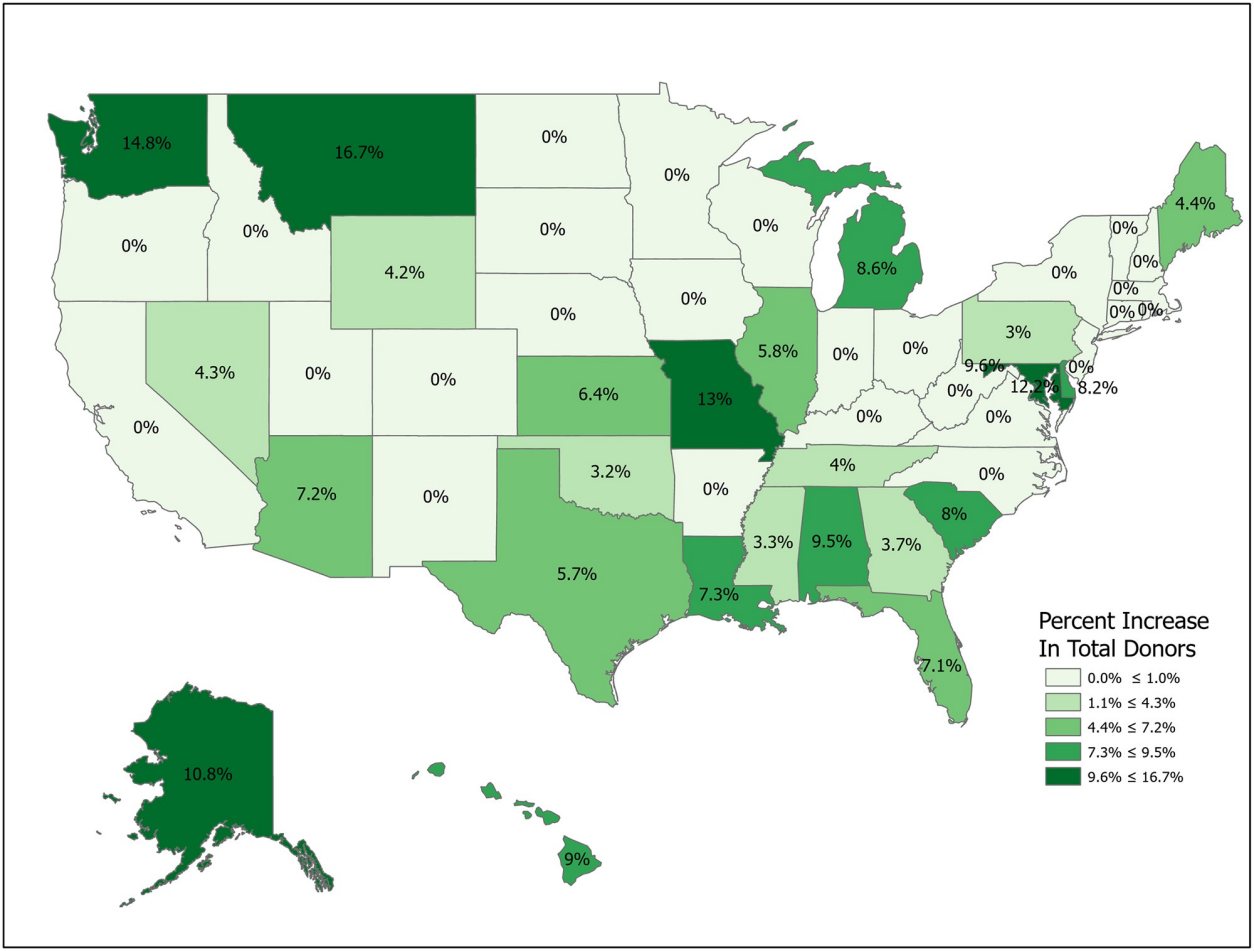
Simulation 5: Target states with high potential donor death rates (*q*_1_). Data come from Table 6. Maps created by ArcGIS Pro, URL: https://www.esri.com/en-us/arcgis/products/arcgis-pro/overview. Base Map: World Light Gray Base, URL: https://services.arcgisonline.com/arcgis/rest/services/Canvas.

**Fig 12:**
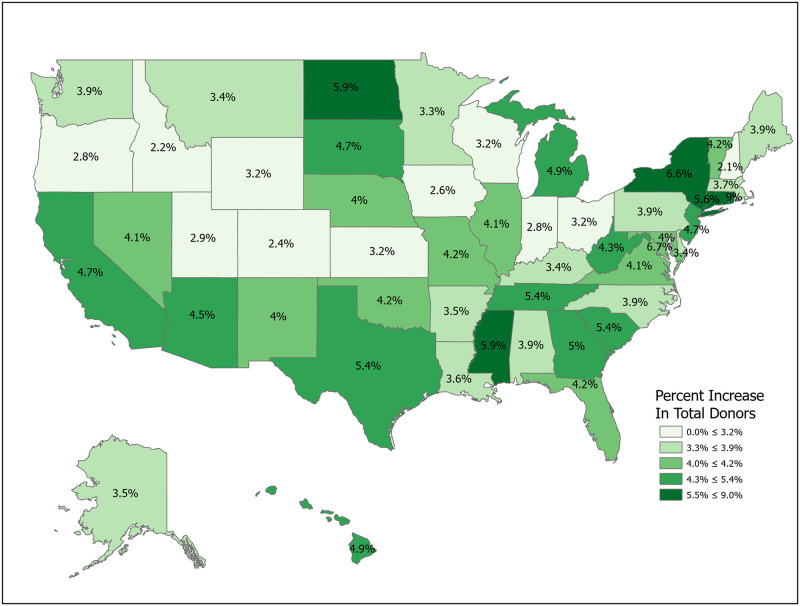
Simulation 6: Increase non-registered donor authorization rate (*q*_2_) by 5 percentage points. Maps created by ArcGIS Pro, URL: https://www.esri.com/en-us/arcgis/products/arcgis-pro/overview. Base Map: World Light Gray Base, URL: https://services.arcgisonline.com/arcgis/rest/services/Canvas.

**Fig 13:**
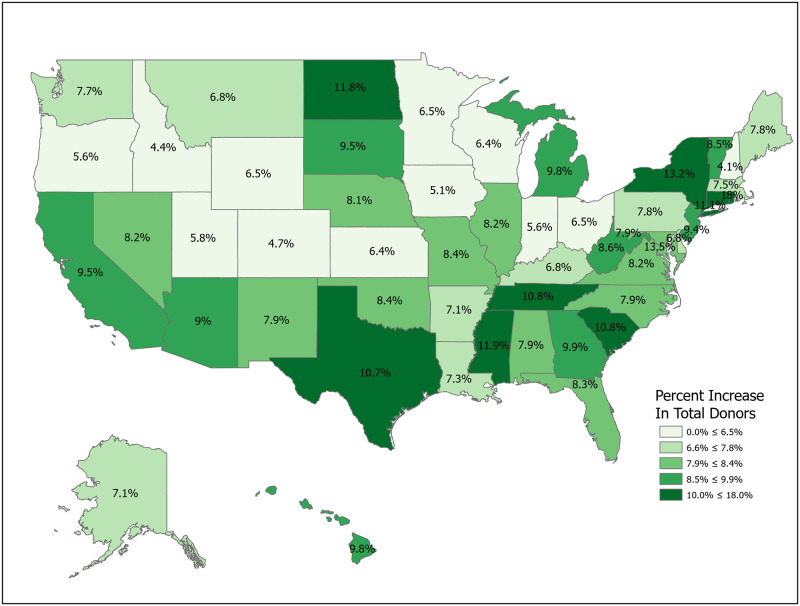
Simulation 7: Increase non-registered donor authorization rate (*q*_2_) by 5 percentage points. Data come from Table 6. Maps created by ArcGIS Pro, URL: https://www.esri.com/en-us/arcgis/products/arcgis-pro/overview. Base Map: World Light Gray Base, URL: https://services.arcgisonline.com/arcgis/rest/services/Canvas.

**Fig 14:**
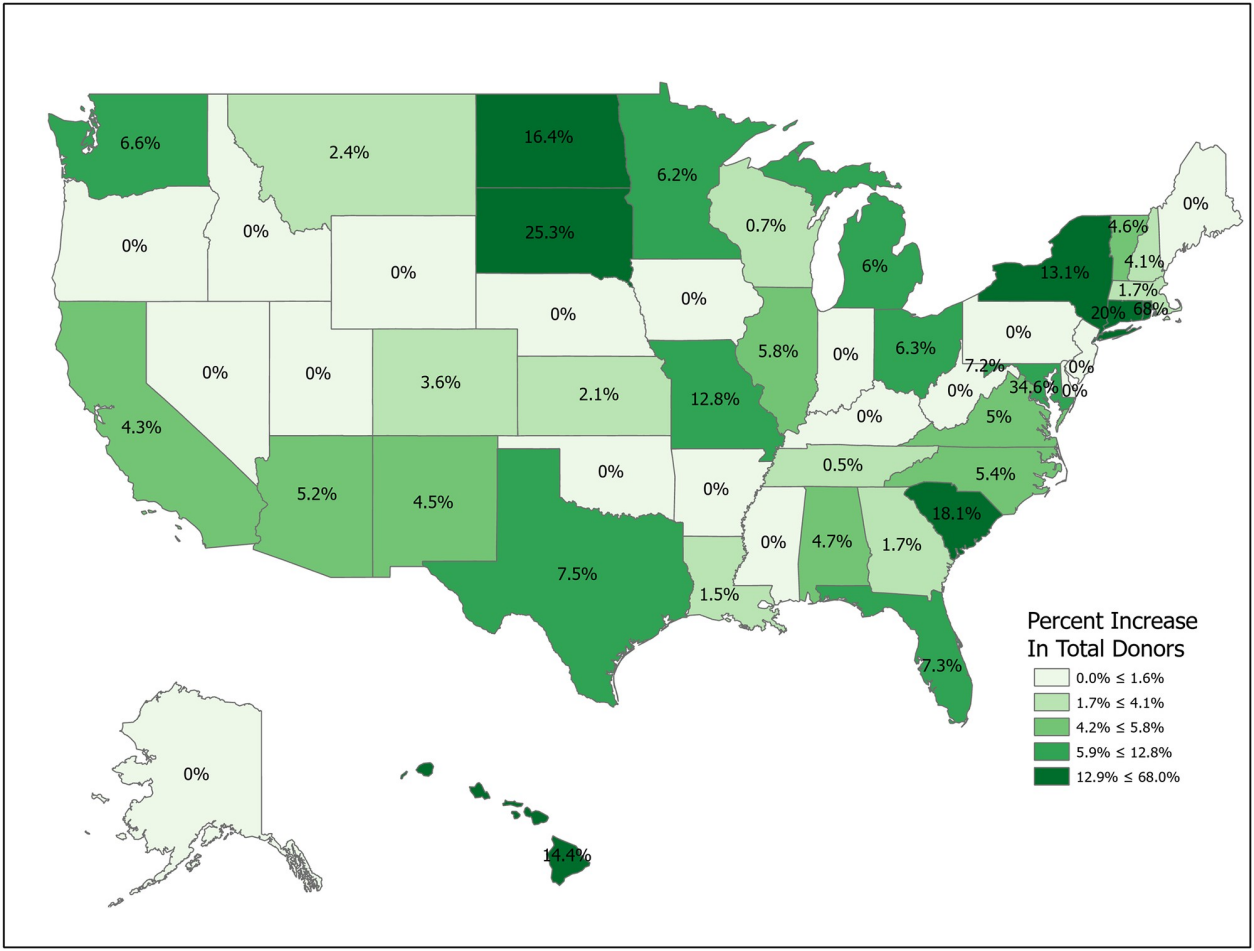
Simulation 8: Raise Non-registered donor authorization rate (*q*_2_) to Minimum of 0.6. Data come from Table 6. Maps created by ArcGIS Pro, URL: https://www.esri.com/en-us/arcgis/products/arcgis-pro/overview. Base Map: World Light Gray Base, URL: https://services.arcgisonline.com/arcgis/rest/services/Canvas.

Simulations 1-5 involve increases in registration rates and are evaluated using Δ, as noted above. Simulation 1 is a uniform, nationwide 10 percent increase in donor registrations. This results in 15,097,334 new donor registrations and an expected increase of 329 donors from a baseline of 10,670. Figs 7–14 illustrate state-level effects of Simulations 1-8. Figs 7–14 were created from our computations using ESRI ARCGIS mapping software [27].

Simulations 2-5 compare the results from Simulation 1 with alternative registration efforts targeting particular sets of states, with the set of states depending on various metrics. For ease of comparison, with these targeted registration simulations we fix the number of new registered donors at 15,097,334, the result from Simulation 1. In each of Simulations 2-5 we raise the number of new registered donors by 20 percent in the targeted states. For example, in Simulation 2 we sort states by their Δ values and then raise registration rates by 20 percent sequentially in each state until we reach a total of 15,097,334 new registrants. Since states are discrete, this implies that we use only a fraction of the last included state. For Simulation 2 this means using the full population of 23 states (Delaware through New York) and a portion of Virginia in order to match the registration number. Simulations 3-5 repeat the exercise for different targeting criteria. The spreadsheets used to perform the calculations are included in the Supplemental Materials. Readers can use the data to perform other simulations as needed.

As mentioned, Simulation 2 targets high Δ states. Using Table 1, this means raising R by 20 percent in Delaware through New York and part of Virginia. This results in an expected increase of 417 donors, an increase of 88 donors (26.7 percent) over the gain from Simulation 1.

Targeting states with low registration rates is also a natural choice. Simulation 3 increases the number of new donor registrations by the same amount as in Simulations 1 and 2, but raises the number of additional donors by only 299. This surprising result highlights the problem of focusing solely on registration numbers to the exclusion of other key variables.

Simulations 4 and 5 follow the same pattern as Simulations 1-3, but focus on other components of Δ. Simulation 4 shows that targeting states with low non-registered Donor Authorization rates (*q*_2_) and high Potential Donor Death rates is more effective than focusing on low registration rates, raising donors by 384 and 406, respectively.

An alternative way to increase actual donors is to raise Donor Authorization rates among the non-registered [6, 8–12]. Methods to accomplish this tend to focus on the training of hospital staff in how to talk persuasively to the families of potential donors. Nationally, the Authorization rate is 56 percent for the non-registered compared with 96 percent among registered donors. For these simulations we use *T* = *Rp*_1_ *p*_2_+ (*M* − *R*)*q*_1_ *q*_2_ (Eq 5) to compute the number of donors and then estimate the change in donors by changing the parameters *p*_2_ and *q*_2_. All estimates are computed at the state level and aggregated.

Donor Authorization rates among the non-registered range widely across states, from 22 percent in Rhode Island to 82 percent in Puerto Rico. Simulation 6 increases *q*_2_ by 5 percentage points, state by state. This raises the number of expected donors by 463. Simulation 7 repeats the exercise, but uses a 10 percentage point increase in *q*_2_. This doubles the change in the number of expected donors to 926. Simulation 8 simulates a targeted effort to raise *q*_2_ to 60 percent in all states that are below that level. This simulation estimates the number of expected donors to increase by 541.

## Discussion

In this paper we present estimates of potential donor death rates and donor authorization rates by registration status. Although our estimates are for the U.S., the approach can be used in any country if modified to fit local definitions and data constraints. We have shown that these probabilities vary markedly across states and by age and gender. We also derive a simple measure of the value of a new registered donor and find that it, too, varies across states and groups.

Our results suggest that there are likely to be significant gains from targeted registration efforts. Since donated organs can move across state lines for transplantation, it seems wise to spend registration resources where they will do the most potential good. As an example, registration funding could be allocated to those states in which potential donor death rates are high and donor authorization rates are low among the non-registered population.

The simulations suggest that for approximately the same cost of recruiting a new donor registrant, between 299 and 926 additional organ donors would result if registrations were targeted more purposefully. Mendeloff *et al*. estimate that for every additional donor, 1.55 kidneys, 0.37 hearts, and 0.76 livers are transplanted. Taking the case of kidneys, this implies from 463 to 1,435 additional kidneys for transplantion. Accounting for the value of all donated organs, they estimate that the net economic value for a new organ donor is approximately $1 million. The simulations therefore imply a potential net economic gain from retargeting to be from about $300 million up to nearly $1 billion.

Our estimates also highlight the importance of using other methods to increase the number of transplants. For example, we have simulated the gains from increasing donor authorization rates. Our estimates suggest that this may be more productive than donor registration efforts. As an example, approximately $1 million dollars a year was spent on promoting donor registries 2010—2012 in the OPO that covers most of West Virginia and part of Pennsylvania [15]. Part of those expenditures could instead be used on staffing and training to increase authorization rates for non-registered potential donors in the same region. Our simulations suggest this might be worth experimenting with.

One weakness of this analysis is that it assumes that registrants are drawn at random from the unregistered population. If, as seems likely, those willing to register are from families that naturally favor organ donation, the registration propensity will be positively correlated with *q*_2_; therefore, the increase in the donor authorization rate (*p*_2_ − *q*_2_) for a new registrant will be smaller than for a randomly-selected unregistered person. In this case Δ should be considered an upper bound. For this reason, we believe that it is more useful to think of Δ as a relative value when comparing across states and subgroups.

We believe that it is worthwhile to pay more attention to the underlying determinants of organ donation rates, especially potential donor death rates, eligible death rates and donor authorization rates for the registered and non-registered populations. The resources used for registration of new donors will be better allocated when policy-makers have better and more precise data. At present we still lack accurate and up-to-date estimates of the demographics of the registered and non-registered.

## Appendix

### Estimating the number of potential donor deaths

Referring to Fig 1, the labels *x*_1_ − *x*_10_ refer the number in each set. We know *x*_5_, *x*_6_, *x*_9_ and *x*_10_ because these are Eligible Deaths listed in the UNOS Eligible Death file. We know *x*_7_ and *x*_8_ because we know the count of donors who are listed in the UNOS Deceased Donor file but who are not in the Eligible Death file. We seek to estimate *x*_3_ and *x*_4_, the numbers of registered and non-registered Potential Donor Deaths who are neither Authorized Donors nor Eligible Deaths.

We assume that the Donor Authorization rates vary only with registration status, not on physical characteristics distinguishing Eligible Death Status. Then Authorization rates are the same for registered donors who are Eligible Deaths and for those who are in the larger group of Potential Donor Deaths. Then our estimate of the Donor Authorization rates among registered and non-registered Eligible Deaths are $\frac{x_{9}}{x_{5}+x_{9}}$ and $\frac{x_{10}}{x_{6}+x_{10}}$, respectively. Donor Authorization rates among all Potential Donor Deaths are $\frac{x_{7}+x_{9}}{x_{3}+x_{5}+x_{7}+x_{9}}$ and $\frac{x_{8}+x_{10}}{x_{4}+x_{6}+x_{8}+x_{10}}$. Given our assumption, these rates for each registration status type are equal. Solving, we have $x_{3}=\frac{x_{5}x_{7}}{x_{9}}$ for the registered and $x_{4}=\frac{x_{6}x_{8}}{x_{10}}$ for the non-registered. So now we have direct measures of *x*_5_ through *x*_10_ and estimates of *x*_3_ and *x*_4_. The remaining values *x*_1_ and *x*_2_ are then estimated using total population numbers. The formulas for the probabilities are given in Table 7 below.

**Table 7:** Data sources and formulas.

Definition	Source/Formula
*Eligible Deaths*, *E* = *x*_5_ + *x*_6_ + *x*_9_ + *x*_10_	STAR Eligible Deaths File, UNOS/OPTN
*Authorized Donor*, *A* = *x*_7_ + *x*_8_ + *x*_9_ + *x*_10_	STAR Deceased Donor File, Eligible Deaths File
*Registered*, *R* = *x*_1_ + *x*_3_ + *x*_5_ + *x*_7_+*x*_9_	Donate Life America 2019 Update
*Non-Registered*, *NR* = *x*_2_ + *x*_4_ + *x*_6_ + *x*_8_+*x*_10_	Donate Life America 2019 Update
$p_{1}=P\left(PD|R\right)=\frac{P(PD\cap R)}{P(R)}$	$p_{1}=\frac{x_{3}+x_{5}+x_{7}+x_{9}}{x_{1}+x_{3}+x_{5}+x_{7}+x_{9}}$
$p_{2}=P\left(A|PD\cap R\right)=\frac{P(A\cap PD\cap R)}{P(PD\cap R)}$	$p_{2}=\frac{x_{7}+x_{9}}{x_{3}+x_{5}+x_{7}+x_{9}}$
$q_{1}=P\left(PD|NR\right)=\frac{P(PD\cap NR)}{P(NR)}$	$q_{1}=\frac{x_{4}+x_{6}+x_{8}x_{10}}{x_{2}+x_{4}+x_{6}+x_{8}+x_{10}}$
$q_{2}=P\left(A|PD\cap NR\right)=\frac{P(A\cap PD\cap NR)}{P(PD\cap NR)}$	$p_{2}=\frac{x_{8}+x_{10}}{x_{4}+x_{6}+x_{8}x_{10}}$
*x*_3_: Assumes *P*(*A*|*PD* ∩ *NR*) = *P*(*A*|*E* ∩ *NR*)	$\hat{x_{3}}=\frac{x_{5}x_{7}}{x_{9}}$
*x*_4_: Assumes *P*(*A*|*PD* ∩ *R*) = *P*(*A*|*E* ∩ *R*)	$\hat{x_{4}}=\frac{x_{6}x_{8}}{x_{10}}$

### Estimation and inference

We seek estimators for the four probabilities and for our statistic, Δ. For Potential Donor Deaths, we assume the probability of an individual becoming an Potential Donor is either *p*_1_ = *P*(*PD*|*R*) or *q*_1_ = *P*(*PD*|*NR*), depending on registration status. The analogous Donor Authorization probabilities are *p*_2_ = *P*(*A*|*PD* ∩ *R*) and *q*_2_ = *P*(*A*|*PD* ∩ *NR*). We assume each individual is an independent Bernoulli trial. For each parameter, denote the trial for individual *i* from a population of size *N* be *X*_*i*_, so that *X*_*i*_∼*B*(*θ*), where *E*(*X*_*i*_) = *θ* and *Var*(*X*_*i*_) = *θ*(1 − *θ*). Then the maximum likelihood estimator $\hat{\theta }$ for the sample mean $\bar{X}$. As usual, $E(\hat{\theta })=\theta$ and $Var\left(\hat{\theta }\right)=\frac{\theta (1-\theta )}{N},$ and $\hat{\theta }$ will be asymptotically normally distributed. That is, ${\hat{q}}_{1}∼N(q_{1},\frac{q_{1}\left(1-q_{1}\right)}{N})$.

Our measure of the expected increase in donors from a new registrant, Δ = *q*_1_(*p*_2_ − *q*_2_), is a non-linear function of three of these parameters. To obtain its variance we use the so-called “delta method”:

$$\begin{array}{c} Var\left(\hat{\Delta }\right)=\text{grad}\Delta \;\Sigma \;\text{grad}\Delta^{\prime } \end{array}$$ (7)

$$\begin{array}{c} =(p_{2}-q_{2}{)}^{2}\;Var\left(q_{1}\right)+q_{1}^{2}\;Var\left(p_{2}\right)+q_{1}^{2}\;Var\left(q_{2}\right), \end{array}$$ (8)

where gradΔ = (*p*_1_ − *q*_2_, *q*_1_, − *q*_1_). We assume zero correlation across estimators so that Σ is a diagonal matrix with the variances of *q*_1_, *p*_1_ and *q*_2_ on the diagonal.

## Supporting information

S1 File (XLSX)
